# Representing bacteria with unique genomic signatures

**DOI:** 10.3389/fdata.2022.1018356

**Published:** 2022-11-16

**Authors:** Diem-Trang Pham, Vinhthuy Phan

**Affiliations:** Department of Computer Science, University of Memphis, Memphis, TN, United States

**Keywords:** metagenomics, Bloom filter, bacteria detection, NGS analysis, k-mers

## Abstract

Classifying or identifying bacteria in metagenomic samples is an important problem in the analysis of metagenomic data. This task can be computationally expensive since microbial communities usually consist of hundreds to thousands of environmental microbial species. We proposed a new method for representing bacteria in a microbial community using genomic signatures of those bacteria. With respect to the microbial community, the genomic signatures of each bacterium are unique to that bacterium; they do not exist in other bacteria in the community. Further, since the genomic signatures of a bacterium are much smaller than its genome size, the approach allows for a compressed representation of the microbial community. This approach uses a modified Bloom filter to store short k-mers with hash values that are unique to each bacterium. We show that most bacteria in many microbiomes can be represented uniquely using the proposed genomic signatures. This approach paves the way toward new methods for classifying bacteria in metagenomic samples.

## 1. Introduction

Metagenomics is the study of analyzing genomes contained in environmental samples. Recent metagenomic studies revealed that the knowledge of the microbial composition in the human gut shows certain complex mechanisms of disorders of human health (Handelsman et al., 2007), such as diverse as diabetes, depression and rheumatoid arthritis. And although the dysbiosis has been proved to link to the gastrointestinal tract (Eloe-Fadrosh and Rasko, 2013), it can be on any exposed surface or mucus membrane, such as the skin or the respiratory system. This variation can impact the human health (Mart́ın et al., 2014). A challenge in metagenomics that is caused by large and complex metagenomic data is the identification and classification of bacteria in microbial communities that consist of thousands or more environmental microbial species (Teeling and Fo, 2012; Sharpton, 2014). A number of approaches have been developed, including alignment reads to reference genomes, analyzing taxonomically informative gene markers, clustering sequences, assembling sequences into genomes and using k-mer based approach. In any approach, it requires a set of reference genomes as a database or an index. In alignment approach, the metagenome sequences (or reads) from the environment are aligned to the reference genome database. In k-mer based approach, an index is created from k-mers of the reference genomes, and this index is used in identification or profiling. While alignment approach has been shown to be accurate, they require large amounts of time and resources. There are many approaches that utilize gene markers or k-mer have been introduced to reduce the running time while still achieving the high accuracy (Lindgreen et al., 2016).

A Bloom filter is a probabilistic data structure that provides very fast membership queries. This useful data structure has been used in several applications in bioinformatics and metagenomics. FACS (Stranneheim et al., 2010) creates a Bloom filter for each reference genome and inserts all k-mers in the filter. Later in query, if a match was found for a k-mer, a match score is computed and it has to surpass a threshold to be classified to a reference genome. BFCounter (Melsted and Pritchard, 2011) introduces an application of Bloom filter to count the k-mers efficiently. BioBloom tool (Chu et al., 2014) applied Bloom filter to create a filter-based sequence-screening tool which was claimed to be faster than BWA, Bowtie 2 and FACS. And another research in building Bloom filters (Pellow et al., 2017) with one-sided k-mers, two-sided k-mers and sparse k-mers data structures improves the performance of the Bloom filter, which will be useful in genome assembly, sequence comparison and sequence search applications. Sequence Bloom Tree (Solomon and Kingsford, 2016), another application of Bloom Filter, is a method for querying thousands of short-read sequencing in RNA-seq experiments for expressed isoforms. This method was able to search large collections of RNA-seq experiments for a given transcript order of magnitude faster than existing approaches.

Most of the existing work use one Bloom filter for each genome, this may not efficiently represent a microbiome or community. In this work, we introduce a method that uses a modified Bloom filter to store unique signatures of bacteria. As such, it can be used to provide unique representation of bacteria in microbiomes. We also show that this method can be used to retrieve species in two microbiomes.

## 2. Methods

Similar to other existing profiling methods, our method consists of two procedures. The first procedure builds an *index* based on the genomes of all the bacteria that might exist in metagenomic samples. The index stores unique genomic signatures of each genome in the microbiome. Once an index is built, it can be used to identify, classify or profile metagenomic samples. Given reads in a metagenomic sample, the second procedure, known as *the querying phase*, makes a query for each read to identify which bacterial genome the read may come from.

### 2.1. Set membership determination with bloom filters

A Bloom filter is a space-efficient probabilistic data structure used for set membership queries. Technically, a Bloom filter is an *M*-bit array *B*, which is initially all zeros, together with a set of *n* hash functions. To prepare a Bloom filter for identifying elements in a universe of elements, each element *x*_*i*_ is hashed to obtain *n* hashed values *h*_1_(*x*_*i*_), ⋯ , *h*_*n*_(*x*_*i*_). Each entry *B*(*h*_*j*_(*x*_*i*_)) is set to 1.

To check whether an item *y* exists in *B*, *n* hash values *h*_1_(*y*), ⋯ , *h*_*n*_(*y*) are computed. If all values are 1, the query answer is True. If not, it is False.

In membership querying, a Bloom filter does not make a false negative. A query to an element in the universe, which is stored in the filter, always correctly returns True. A false positive, however, can happen. Due to the nature of simply setting all hashed entries to 1 in the filter building phase, it is possible that the query of an element *z* that is not stored in the filter actually returns True. It is known that to minimize the probability of getting false positives, the optimal number of hash functions should be $\frac{b\ln\;2}{m}$, where *b* is the size (number of bits) of the filter, and *m* is the number of elements stored in the filter (Bloom, 1970).

### 2.2. Finding k-mers with genome-unique hash values

Given a set of referenced bacterial genomes that might exist in the metagenomic environment of interest, an index, *F*, which is a modified Bloom filter, is built to store unique genomic signatures of each genome.

The index, *F*, is an array with *m* entries. During the processing of referenced genomes, k-mers from these genomes are hashed into *F* using *n* randomly generated hash functions. A k-mer *x* is hashed into *n* entries *h*_1_(*x*), ⋯ , *h*_*n*_(*x*) of *F*. After all referenced genomes are processed, an entry of *F* with a positive value *g* corresponds to a k-mer, whose hash values are unique to genome *g*. This allows *F* to be used in ways similar to those of a Bloom filter to detect genomes that are present in the metagenomic sample. The construction of *F* consists of two main phases. In each phase, all genomes are sequentially processed by Algorithm 1. In both phases, Algorithm 1 shares a common goal: it attempts to identify k-mers with hash values that are unique to the genome. It does this by going through each k-mer of the genome and marking all *n* locations (determined by *n* hash values) with dirty or with the genome id. A location is dirty (set to -1) if two k-mers on two different genomes get hashed to it. If a location is not dirty, it stores the id of some genome. If a k-mer *x* of genome *g*_1_ is hashed to an entry that holds the id of another genome, say *g*_2_, then that *x* is not unique and all entries *h*_1_(*x*), ⋯ , *h*_*n*_(*x*) of *F* are set to dirty. If *x* is deemed unique, the genome id is stored in all of these entries. Suppose that after Phase 1, genomes *g*_1_, ⋯ , *g*_*l*_ are processed sequentially in this order. Entries in *F* with values *g*_1_ may not correspond to k-mers with unique hash values. To see this, suppose k-mer *x* appears in both *g*_1_, k-mer *y* appears in *g*_2_, and some of the hash values of *x* and *y* are the same. Because *g*_2_ is processed after *g*_1_, all the entries corresponding to the hash values of *y* are set to dirty, but not all the entries corresponding to the hash values of *x* are set to dirty.

**Algorithm 1:** ProcessGenome(*F*, *gid*, *phase*).


1: *positions* = []
2: **for** k-mer *k* at position *pos* in genome *gid* **do**
3: *unique* = *True*
4: *idx* = []
5: **for** each hash function *f* **do**
6: *v* = *f*(*k*)
7: *idx*.*append*(*v*)
8: **if** *F*[*v*]≠0 **and** *F*[*v*]≠*gid* **then**
9: *unique* = *False*
10: **if** *unique* **then**
11: **if** *phase* == 2 **then**
12: *positions*.*append*(*pos*)
13: **for** each value *v* in *idx* **do**
14: *F*[*v*] = *gid*
15: **else**
16: **for** each value *v* in *idx* **do**
17: *F*[*v*] = −1
18: **if** *phase* == 2 **then**
19: *Reduce*(*F*, *gid*, *positions*)

It is, however, important to understand that after Phase 1, entries in *F* with values *g*_*l*_ will in fact correspond to k-mers in genome *g*_*l*_ with hash values unique to this genome. Since *g*_*l*_ is processed last, if an entry in *F* has value *g*_*l*_, it means some k-mer in *g*_*l*_ with hash values that do not collide with any k-mer in all the other genomes that are already processed. Thus, this k-mer has hash values that are unique; no other k-mer in any other genome shares one of these hash values. Therefore, when a genome is processed by Algorithm 1 after all of the other genomes have already been processed, all of k-mers with unique hash values in that genome are correctly marked in *F*. This means that after Phase 2, when all genomes are processed again by Algorithm 1, all k-mers with unique hash values in all genomes will be correctly marked in *F*.

**Algorithm 2:** Reduce(*F*, *gid*, *positions*).


1: *selected* = [*positions*[0]]
2: **for** *i* = 1; *i*<*len*(*positions*); *i* = *i*+1 **do**
3: **if** *selected*[*len*(*selected*)−1]+ω < *positions*[*i*] **then**
4: *selected*.*append*(*positions*[*i*])
5: **else**
6: Let *x* be the k-mer at *positions*[*i*] in genome *gid*
7: **for** each hash function *f* **do**
8: *F*[*f*(*x*)] = −1
9: **for** each position *p* in *selected* **do**
10: Let *x* be the k-mer at *p* in genome *gid*
11: **for** each hash function *f* **do**
12: *F*[*f*(*x*)] = *gid*

### 2.3. Query phase: Reads processing

Given reads from a metagenomic sample, the main task is to identify which bacteria exist in the sample. This boils down to processing reads and determining which bacterial genomes they most likely belong to. While all existing methods we are aware of process all reads in the metagenomic samples, the proposed method processes just enough reads to cover a fraction of bacterial genomes. This typically results in choosing a small random samples of reads for processing.

If a processed read belongs to a genome *g* and also contains a k-mer *x* with unique hash values stored in *F*, there is a good chance that the read will be correctly identified to belong to *g*. The read is not recognized if the k-mer *x* has a sequencing error or a genetic variant. A genetic variant can occur because the genome of the bacterium in the sample is likely not the same as the referenced genome of the same bacteria used to create *F*.

A processed read that does not belong to genome *g* might also be mistakenly identified to belong to *g* if it has a sequencing error or a genetic variant that results in a k-mer with hash value(s) collide with one of the k-mers of *g* stored in *F*.

Given a read to be processed, all k-mers are passed into k-mer processing to classify its *g*_*i*_. Let *V* be the set of classified *g*_*i*_ of all k-mers of the read. If *V* consists of only 0 and/or –1, then the read is discarded. If, however, *V* consists of positive values, i.e., genome ids, then one of three different strategies can be used to determine which genome the read belongs to.

#### 2.3.1. Majority

If there is a positive number, *g*, in *V* with frequency greater than 50%, then *g* is predicted to be the genome that contains the read. If there is no such number, then the read is discarded. This strategy is effective in the presence of significant amounts of sequencing errors and/or genetic variants. In such cases, a k-mer of the read can be misidentified to be a unique k-mer of a different bacteria. But if there are not too many of such mistakes, a majority of positive identification can identify the correct genome.

#### 2.3.2. First-hit

K-mers are processed sequentially. When the first k-mer that has a positive hash value, *g*, is encountered, no additional k-mers are processed. *g* is predicted to be the genome that contains the read. This strategy is effective when k-mers stored in *F* are highly unique so that the first hit is most likely correct.

#### 2.3.3. One-or-nothing

If *V* has only one positive value, *g*, then *g* is predicted to be the genome that contains the read. If this is not the case, the read is discarded. This strategy is highly conservative. If there is a disagreement, i.e., two genomes identified by different k-mers of the reads, the read is discarded from consideration.

**Figure 1:**
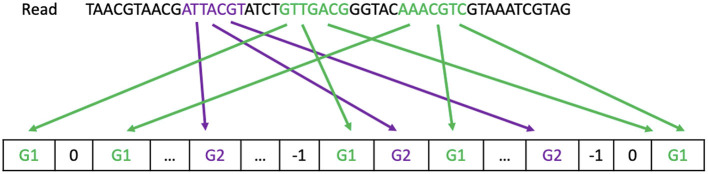
Read processing. The majority strategy predicts the read comes from G1. The First-hit strategy predicts the read comes from G2. The One-or-nothing strategy discards the read.

Figure 1 gives an example on how each strategy classifies a read to a reference genome.

In order to optimize the running time of query phase, reads are distributed to different cores for processing.

## 3. Results

### 3.1. Experimental setup

To assess performance of our method, we used two microbial communities, with included 457 and 2,850 reference genomes, respectively. The first community consists of 457 reference genomes, named S1, combined from three metagenomes used by Mende et al. (2012) in a study of metagenomic assembly. To create a set of reference genomes, we extracted accession numbers from reads in these three metagenomes. This information allowed us to retrieve from NCBI reference genomes for the bacteria, from which the reads were created. The second community, named S2, includes genomes used in CAMI challenge (Sczyrba et al., 2017).

First, we show some statistics of the indexes of each reference genome set. Second, we compare results on different querying strategies. And finally, we also show the difference of indexes when using different number of hash functions.

### 3.2. Representing bacteria using unique signatures

We now report how the two microbial communities can be represented by unique genomic signatures. For the first set of bacterial genomes S1, we used 2 hash functions, k-mer of length 31 and the size of the index is 8GB. The index was built in two phases. All 457 genomes have unique signatures. Total number of signatures is 248,758,006. Minimum number of unique signatures is 152 and maximum number of unique signatures is 1,720,014.

**Figure 2:**
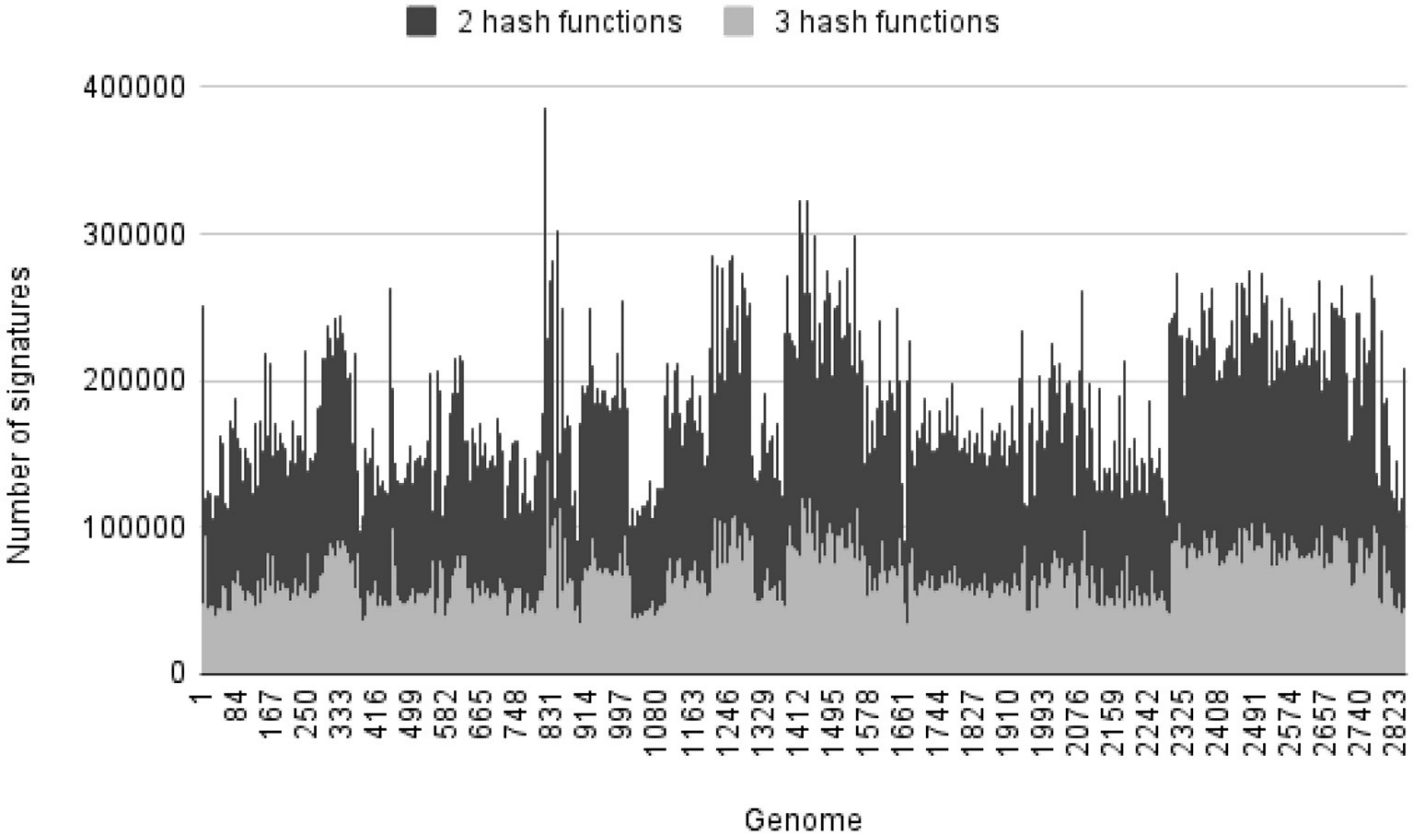
Number of unique signatures for each genome in genome set S2 in the change of number of hash functions.

As the more hash functions are used in building index, the more hash values are computed for each k-mer and the more unique it is. But that will also reduce the number of k-mers with unique hash values for each genome. Although larger genomes have a sufficient number of k-mers with unique hash values, smaller genomes have only a few of such unique k-mers. For this bacterial genome set S2, we build two indexes with the same k-mer size and index size, and only vary the number of hash functions to compare the effect on querying performance when different number of hash functions were used to build the index. Both indexes are built in 1 phase. All the genomes have unique signatures. Table 1 shows the total, the minimum, and the maximum number of signatures of each index. We found that the 3-hash-function index had fewer signatures than the 2-hash-function index. This is likely because as more hash values were computed, there was a higher chance of having collisions of those hash values. Figure 2 shows the distribution of number of unique signatures for each genome in genome set S2 in the change of number of hash functions when building index for set S2.

**Table 1:** Comparison on number of signatures in the change of number of hash functions when building index for set S2.

**Number of hash functions**	**Min**	**Max**	**Total**
2	544	386,709	400,769,054
3	211	145,005	150,366,923

### 3.3. Querying

In order to evaluate the retrieval capability of our two indexes, we downloaded two simulated samples for querying.We use the 10 species dataset by Mende et al. (2012) which consists of genomic reads from 10 genomes in S1, and the RH_S001 dataset from Sczyrba et al. (2017) consists of 302 genomes from S2. We will refer to these datasets as 10 species and RH_S001 in subsequent discussions. Reads from 10 species and RH_S001 are paired-end and were simulated with charateristics of Illumina sequencing technology with length of 75 and 150 bp, respectively. The 10 species dataset is used to query bacteria in S1, and the RH_S001 is queried in S2.

Performance was measured in terms of precision, recall and F1-score as accuracy of the predicting process. Precision is computed as the number of correctly queried bacteria divided by the total number of predicted bacteria. Recall is the number of correctly queried bacteria divided by the total number of bacteria that actually exist in the sample. F1-score is the harmonic mean of precision and recall.

The 10 species sample is queried on index of set S1 using majority strategy. We were able to query all 10 species, results in recall of 100%. However, there are many incorrect querying, this leads to low precision of 2.6%. The F1-score is 5%. We also evaluated the performance of different querying strategies. As described earlier, the majority query strategy looks at all k-mers and picks the genome that shows up at least 50% among all k-mers. The one-or-nothing query strategy picks a genome only if it is the only genome predicted by all k-mers of the read. The first-hit strategy picks the first genome that is predicted by some k-mer of the read. Each of these strategies has its own pros and cons. And the most appropriate strategy depends on the dataset. Table 2 shows the performance resulted from each of the three query strategies.

**Table 2:** Effect of different number of querying strategies.

**Query strategy**	**Precision**	**Recall**	**F1-score**
Majority	0.026	1.000	0.051
First-hit	0.026	0.990	0.051
One-or-nothing	0.028	0.987	0.053

We found that the performance resulted from the three query strategies was very similar. Both majority and first-hit strategies had lower precision, but higher recall than one-or-nothing. One-or-nothing, by design, is more conservative, and therefore, should have fewer false positives, and higher precision than the other two strategies.

The RH_S001 sample is queried on the index of set S2 using the majority strategy. There are 162 out of 302 genomes correctly predicted. Only 5 genomes in the sample are missing as there may have sequencing errors in the reads that causes wrong prediction to other genomes. Another reason is that no read has the exact unique signatures in the index. This leads to a precision of 26%, recall of 97% and F1-score is 41%.

Table 3 shows the effect on querying performance when two or three hash functions were used to build the index. We found that using 2 hash functions to build an index resulted in a slightly better overall performance than using 3 hash functions. While recall rates were similar, precision rates were higher when 2 hash functions were used. In this experiment, we used the majority strategy, and having more signatures could be useful for this querying strategy to reduce the false positive, which improves the precision.

**Table 3:** Effect of different number of hash functions.

**Number of hash functions**	**Precision**	**Recall**	**F1-score**
2	0.316	0.601	0.414
3	0.296	0.601	0.396

## 4. Discussion

We introduced a method for representing bacteria in a microbial community uniquely. We showed that our method could be used to query reads in metagenomic samples. A method for efficiently representing bacteria in a microbial community would be useful for post-processing in order to have an accurate identification of bacteria, which requires more analysis as well as data interpretation on the query outputs. And due to the close relationship between the microbiome and health, improving the accuracy of bacteria identification would help to make metagenomic analysis more meaningful in understanding the human microbiome in health and disease. There is room to find parameters that can improve the performance of the query phase. Also, additional improvements can be made in the future to determine these choices more appropriately under different criteria.

Similar to most of other k-mer based approaches, when the database consists of hundreds of thousands reference genomes, it is challenging for the proposed method to obtain unique signatures for some genome, especially very small genomes. This method, however, can be promising for microbiomes that are not too big, e.g., skin, oral, or gut microbiomes.

## Data availability statement

Publicly available datasets were analyzed in this study. This data can be found at: illumina 10 species http://www.bork.embl.de/~mende/simulated_data/; High complexity sample 1 https://edwards.flinders.edu.au/cami-challenge-datasets/.

## Author contributions

D-TP and VP designed the methods and experiments and wrote the paper. D-TP wrote code, downloaded data, and ran experiments. Both authors contributed to the article and approved the submitted version.

## Conflict of interest

The authors declare that the research was conducted in the absence of any commercial or financial relationships that could be construed as a potential conflict of interest.

## Publisher's note

All claims expressed in this article are solely those of the authors and do not necessarily represent those of their affiliated organizations, or those of the publisher, the editors and the reviewers. Any product that may be evaluated in this article, or claim that may be made by its manufacturer, is not guaranteed or endorsed by the publisher.
